# Role of procalcitonin as an early market in diagnosis and follow up of surgical site infection in Al Azhar University Hospital – New Damietta, Egypt

**DOI:** 10.1186/2047-2994-4-S1-P85

**Published:** 2015-06-16

**Authors:** M Abdelnasser, M Gohar, M Saleh, M Abdelhamid, M Balboula

**Affiliations:** 1 Microbiology & Immunology, Faculty of Medicine, Al-Azhar University, Cairo, Egypt; 2 Surgery, Faculty of Medicine, Al-Azhar University, Cairo, Egypt

## Introduction

Identifying patients with bacterial infection and sepsis is a major challenge in emergency departments and critical care units. Procalcitonin (PCT), the prohormone of calcitonin, was described as innovative parameter in early diagnosis of infection.

## Objectives

This work was carried out to evaluate the role of PCT as an early marker in diagnosis and follow up of patients with surgical site infection.

## Methods

The study was conducted on 50 patients admitted in the surgical departments of New Damietta, University Hospital, in the period between September 2012 and September 2013. Blood, urine and/or pus cultures were done. White blood cell (WBC) counts were determined using the automated hematology analyzer. Serum C-reactive protein (CRP) level was measured by the semi-quantitative latex agglutination test. Serum PCT concentrations were determined using the enzyme linked immunosorbent assay (ELISA).

## Results

Postoperative infections were found in 29 patients (58 %) with SSI in 11 (22 %), urinary tract infection (UTI) in 5 (10 %), blood stream infection (BSI) in 4 (8 %), SSI and BSI in 7 (14 %) and SSI and UTI in 2 (4 %) of these patients. *Staphylococcus aureus & coagulase negativeStaphylococci* were the most frequently isolated pathogens, followed by *Escherichia coli*, *Pseudomonas aeruginosa*, *Proteus mirabilis*, *Candida albicans*, *Salmonella Paratyphi* B and *Klebsiella pneumoniae*. Most of the isolated organisms were sensitive to *Imipenem.* Among the infected group, median serum levels of PCT and Temperature were higher (P <0.001) in the early phase (one day after operation, 1.20 ng/m,36.5 ºC) more than preoperative phase (0.11 ng/ml, 37.7ºC) and the late phase (5 days after operation: 0.46 ng/ml, 37.5 ºC). Median serum levels of CRP and WBC were increased gradually (P <0.001) from the base line to the late phase (5.0 mg/l, 12.0 mg/l, 48.0 mg/l for CRP and 5.3 K/uL,10.1K/uL, 11.2 K/uL for WBC, respectively).

## Conclusion

These data indicate that PCT is greatly helpful to distinguish an infection from an inflammation. Moreover, it plays a very important prognostic role in the early detection of patients at risk of infection in the post-operative period.

## Disclosure of interest

M. Abdelnasser Employee of: Al-Azhar University, M. Gohar Employee of: Al-Azhar University, M. Saleh Employee of: Al-Azhar University, M. Abdelhamid Employee of: Al-Azhar University, M. Balboula Employee of: Al-Azhar University.

